# Pericardial patch venoplasty heals via attraction of venous progenitor cells

**DOI:** 10.14814/phy2.12841

**Published:** 2016-06-28

**Authors:** Hualong Bai, Mo Wang, Trenton R. Foster, Haidi Hu, Hao He, Takuya Hashimoto, Jesse J. Hanisch, Jeans M. Santana, Ying Xing, Alan Dardik

**Affiliations:** ^1^ The Vascular Biology and Therapeutics Program and the Department of SurgeryYale University School of MedicineNew HavenConnecticut; ^2^ Basic Medical College of Zhengzhou UniversityHenanChina; ^3^ Department of Vascular SurgeryFirst Affiliated Hospital of Zhengzhou UniversityHenanChina; ^4^ Department of Vascular SurgeryThe Second Xiangya Hospital of Central South UniversityChangshaChina; ^5^ Department of SurgeryVA Connecticut Healthcare SystemWest HavenConnecticut

**Keywords:** Aortocaval fistula, arterioplasty, arteriovenous fistula, AVF ligation, Eph‐B4, pericardial patches, pseudoaneurysm, venoplasty, venous neointimal hyperplasia

## Abstract

Pericardial patches are commonly used during cardiovascular surgery to close blood vessels. In arteries, patches accumulate arterial progenitor cells; we hypothesized that venous patches would accumulate venous progenitor cells, in the absence of arterial pressure. We developed a novel rat inferior vena cava (IVC) venotomy model and repaired it with a pericardial patch. Cells infiltrated the patch to form a thick neointima by day 7; some cells were CD34^+^/VEGFR2^+^ and CD31^+^/Eph‐B4^+^ consistent with development of venous identity in the healing patch. Compared to arterial patches, the venous patches had increased neointimal thickness at day 7 without any pseudoaneurysms. Addition of an arteriovenous fistula (AVF) to increase blood flow on the patch resulted in reduced patch neointimal thickness and proliferation, but neointimal thickness was not reversible with AVF ligation. These results show that rat patch venoplasty is a novel model of aggressive venous neointimal hyperplasia.

## Introduction

Pericardial patches, either bovine or porcine, are commonly used by surgeons to close blood vessels during cardiovascular surgery (Muto et al. 2009; Li et al. 2011). Biasi et al. showed that pericardial patch angioplasty reduced the rate of carotid artery restenosis compared to primary closure (Biasi et al. 2002); recent series show long‐term patch stability and freedom from infection and as well as 98% freedom from restenosis in treated carotid arteries (Papakostas et al. 2014). Closure of veins such as the inferior vena cava (IVC) with a pericardial patch is also performed in some cases, including oncology resections (Del Campo and Konok 1994; Ohwada et al. 1999) and living donor liver transplantation (Mori et al. 2012).

Recent studies from our laboratory suggest that pericardial patches may be a unique microenvironment after implantation into an artery; arterial patches attract progenitor cells including arterial progenitor (CD34 and Ephrin‐B2 dual‐positive) cells and endothelial progenitor (CD34 and VEGFR2 dual‐positive) cells, suggesting that the patch forms a microenvironment favorable for accumulation of these cells, allowing this site to acquire an arterial identity (Li et al. 2012). Despite the healing of arterial patches with a mature neointima and infiltration of M2 macrophages (Bai et al. 2016), the rat patch angioplasty model is limited by the development of pseudoaneurysms at longer times, which is likely due to the microsurgical suturing of the patch in the arterial environment (Bai et al. 2016).

The accumulation of progenitor cells in the patch neointima and body after patch angioplasty may be due to specific accumulation of cells secondary to the environment, or to the general accumulation of cells secondary to arterial pressure. We hypothesized that progenitor cell accumulation in the patch is due to the specific patch microenvironment attracting these cells, and not as an epiphenomenon of arterial pressure. To test this hypothesis we developed a rat patch venoplasty model in which pericardial patches are implanted into the IVC, a low pressure, high flow vessel; we hypothesize that venous patches will attract venous progenitor cells, even in the lower venous pressure environment. In addition, we hypothesize that the low pressure of the venous environment will reduce pseudoaneurysm formation in venous patches, confirming that pseudoaneurysm formation in the rat patch angioplasty model is a consequence of arterial pressure.

## Materials and Methods

### Animal model

All experiments were approved by the Institutional Animal Care and Use Committee at the Yale University School of Medicine. Male Wistar rats (6–8 week old) were used for patch implantation (*n* = 43) as previously described (Li et al. 2012). Microsurgical procedures were performed aseptically in a dedicated facility, using a dissecting microscope (Leica MZ 95, Germany). Anesthesia was given via isoflurane inhalation. A midline incision was made in the abdomen, and the infrarenal vena cava (IVC) was exposed. The site of patch implantation was approximately 2 mm below the level of the origin of the renal veins; the IVC was dissected free at this site, and all lumbar veins at this level were ligated and divided using 6‐0 nylon sutures. The infrarenal IVC was clamped and a longitudinal 3 mm venotomy was made on the anterior wall of the IVC. The venotomy was closed with a pericardial patch (3 mm × 1.5 mm × 0.6 mm; Xenosure; LeMaitre Vascular, Burlington, MA) using interrupted 10‐0 nylon sutures. After completion of the venoplasty closure, the clamps were removed to vent the patch and then restore blood flow in the IVC. The abdomen was then closed and the rat allowed to recover. Aortic patches were implanted in a similar fashion, using the aorta instead of the IVC, as previously described (Li et al. 2012). Subcutaneous patches were implanted in the abdomen.

In some rats, an AVF was also created immediately after patch implantation using an aortocaval fistula as previously described (Yamamoto et al. 2013). In these rats, IVC flow was restored for approximately 5 min after patch implantation; then the infrarenal aorta was exposed and clamped. Just above the aortic bifurcation, a 20‐gauge needle was used to puncture the aorta through both walls into the IVC and then immediately removed; a 10‐0 suture was used to close the aortic puncture site and then the aortic clamp was removed and the abdomen was closed.

In some rats, the AVF was ligated at day 7. In these rats, the rat was reanesthetized and the abdomen was reopened, the aorta and IVC were dissected apart to locate the AVF and then the AVF was carefully ligated using a 6‐0 nylon suture, and the abdomen was closed.

Rats were sacrificed on postoperative days 1, 3, 7, 14, or 30 and the patches were explanted for analysis as described below. No immunosuppressive agents, antibiotics or heparin were given at any time.

### Histology

Rats were anesthetized with isoflurane inhalation, and tissues were fixed by transcardial perfusion of phosphate buffered saline (PBS) followed by 10% formalin. The tissue was removed and fixed overnight in 10% formalin followed by a 24‐h immersion in 70% alcohol. The tissue was then embedded in paraffin and sectioned (5 *μ*m thickness). Tissue sections were deparaffinized and stained with hematoxylin and eosin. Neointimal and adventitial thickness were the mean of measurements from the surface edge to the edge of the patch in three independent areas.

### Immunohistochemistry

Tissue sections were deparaffinized and then incubated using primary antibodies overnight at 4°C. After overnight incubation, the sections were incubated with EnVision reagents for 1 h at room temperature and treated with Dako Liquid DAB Substrate Chromogen System (Dako). Finally, the sections were counterstained with Dako Mayer's Hematoxylin.

### Immunofluorescence

Some tissue sections were deparaffinized and immediately examined under the immunofluorescence microscope directly. Otherwise, tissue sections were deparaffinized and then incubated with primary antibodies overnight at 4°C. To visualize and quantify cells, sections were stained with the fluorescent dye 4′, 6‐diamidino‐2‐phenylindole (DAPI; Invitrogen) to mark cellular nuclei. The mean integrated optical density (IOD) of the immunoreactive signals was analyzed, using Image‐Pro Plus 6.0 software (Media Cybernetics; Rockville, MD). The density of rhodamine immunofluorescence was analyzed using ImageJ (National Institutes of Health).

### Western blot

Pericardial patches were carefully harvested and removed from the surrounding tissue; the neointima was carefully dissected free from the patch and snap frozen in liquid nitrogen. Samples were crushed and mixed with buffer including protease inhibitors (Roche, Complete Mini 12108700) prior to sonication (5 sec) and centrifugation (17,000 *g*, 15 min). Equal amounts of protein from each experimental group were loaded for SDS‐PAGE, followed by Western blot analysis with signals detected using the ECL detection reagent. Patches were analyzed individually, without combination of samples.

### Primary and secondary antibodies

Primary antibodies included: anti‐*α*‐actin (Abcam, ab5694; IHC and IF, 1:100; WB, 1:1000); anti‐cleaved Caspase‐3 (Cell Signaling #9661; IHC, 1:50; WB, 1:1000); anti‐CD31 (Abcam, ab28364; IHC and IF, 1:50); anti‐CD34 (R&D, AF4117; IF, 1:100; WB, 1:1000); anti‐CD45 (Abcam, ab10558; IHC, 1:50; WB, 1:1000); anti‐CD68 (ED1; Abcam, ab31630; IHC, 1:200; WB, 1:1000); anti‐ COUP‐TFII (Novus biologicals, NBP1‐67885; IHC, 1:100); anti‐Eph‐B4 (Santa Cruz, sc‐5536; IF, 1:50); anti‐dll‐4 (santa cruz, sc‐18640, 1:50); anti‐Eph‐B4 (Abcam, ab76657; IHC and IF, 1:50); anti‐Ephrin‐B2 (Novus, NBP1‐48610; IHC, 1:50); anti‐Eph‐B4 (R&D, AF446; WB, 1:1000); anti‐GAPDH (Cell Signaling, 14C10; WB, 1:2000); anti‐Ki67 (Abcam, ab15580; IHC, 1:100); anti‐proliferating cell nuclear antigen (PCNA) (Dako monoclonal mouse Anti‐PCNA; IHC and IF, 1:100; WB, 1:1000); anti‐VEGFR2 (ABCAM, ab2349; IF, 1:100; WB,1:1000); and anti‐vimentin (Abcam, ab8978; IHC, 1:50; WB, 1:1000).

Secondary antibodies used for IF were: donkey anti‐goat Alexa‐Fluor‐488, donkey anti‐rabbit Alexa‐Fluor‐488, donkey anti‐rabbit Alexa‐Fluor‐568, donkey anti‐mouse Alexa‐Fluor‐568 and chicken anti‐mouse Alexa‐Fluor‐488 conjugated antibodies from Invitrogen (1:1000). For IHC, sections were incubated with EnVision reagents for 1 h at room temperature and treated with Dako Liquid DAB+ Substrate Chromogen System (Dako). Finally, the sections were counterstained with Mayer's hematoxylin.

### Statistical analysis

Data are expressed as the mean ± SEM. Statistical significance for these analyses was determined by ANOVA and *t*‐tests (Prism 6; GraphPad Software, La Jolla, CA). *P*‐values less than 0.05 were considered significant.

## Results

### Cell infiltration into the venous patch

**Figure 1:**
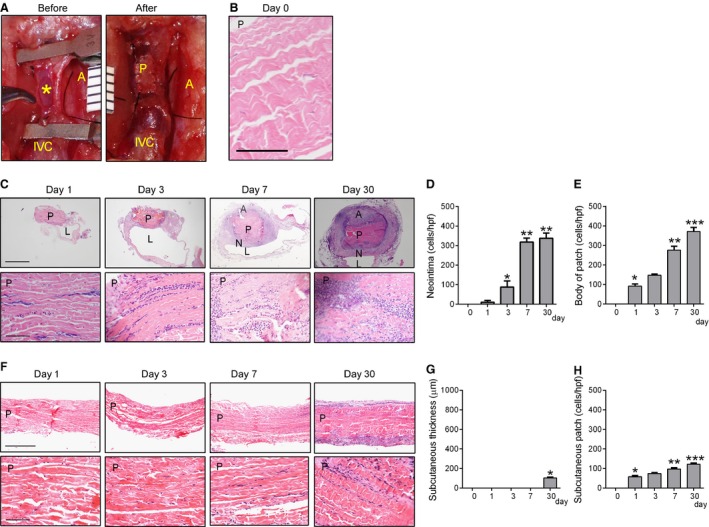
Increased cell infiltration after patch venoplasty. (A) Operative photograph of the inferior vena cava (IVC) before and after patch venoplasty; *, posterior intima of IVC; A, aorta; IVC, inferior vena cava; P, patch; ruler markings are 1 mm. (B) H&E staining of the patch before implantation; P, patch; scale bar, 100 *μ*m. (C) Photomicrographs of patch venoplasty on postoperative days 1–30. Upper row, low power (scale bar, 1 mm); lower row, high power (scale bar, 100 *μ*m). Patches harvested postoperative day 1, 3, 7 or 30 (*n* = 4) and stained with Hematoxylin and eosin. P, patch; L, IVC lumen; A, adventitial (peritoneal) surface; N, neointima. (D) Bar graph showing increased neointimal cells (mean number of cells counted in 4 high power fields); *P* < 0.0001, ANOVA. *P = 0.0125 versus day 1; **P < 0.0001 versus day 3 (post hoc test); *n* = 4. (E) Bar graph showing increased cells in the patch (mean number of cells counted in 4 high power fields); *P* < 0.0001, ANOVA. *P = 0.003 versus day 0; **P = 0.0001 versus day 3; ***P = 0.0021 versus day 7 (post hoc test); *n* = 4. (F) Photomicrographs of patches implanted in the subcutaneous tissue in paired animals. Upper row, low power (scale bar, 500 *μ*m); lower row, high power (scale bar, 100 *μ*m). Patches harvested postoperative day 1, 3, 7 or 30 (*n* = 4) and stained with Hematoxylin and eosin. P, patch. (G) Bar graph showing thickness of tissue surrounding subcutaneously implanted patches; *P* < 0.0001, ANOVA. *P < 0.0001 versus day 7 (post hoc test). *n* = 4. (H) Bar graph showing number of cells in the subcutaneously implanted patches (mean number of cells counted in 4 high power fields); *P* < 0.0001, ANOVA. *P = 0.0001 versus day 0; **P = 0.0027 versus day 1; ***P = 0.0008 versus day 3 (post hoc test). *n* = 4.

To determine the response to patch implantation in the low pressure venous circulation, we developed a model of patch venoplasty in the rat IVC; pericardial patches were sewn into the rat IVC using standard microsurgical techniques (Fig. 1A). Prior to implantation, the patch is composed of collagen fibers without any cells (Fig. 1B). After implantation, patches were incorporated into the IVC wall without any thrombus or pseudoaneurysm formation (Fig. 1C); at days 1 and 3 there were some cells attached to the luminal side of the patch without any neointima formation; similarly, there was also no obvious new tissue formed on the outer “adventitial” surface of the patch. At day 7, there was a distinct layer of neointima forming on the luminal side of the patch with spherical‐shaped cells; there was also a layer of tissue on the adventitial surface adjacent to the peritoneum (Fig. 1C). More cells infiltrated into the neointima overlying the patch over time, with significantly more cells at days 7 and 30 compared to day 3 (Fig. 1D); similarly, there were increased numbers of cells infiltrating into the body of the patches over time (Fig. 1E). In contrast, patches that were simultaneously implanted subcutaneously had less cell infiltration into the patch and only a thin capsule of cells by day 30 (Fig. 1F–H). Immunohistochemistry of these subcutaneous patches showed only a small number of macrophages and smooth muscle cells (data not shown).

### Cell composition of the venous patch neointima

**Figure 2:**
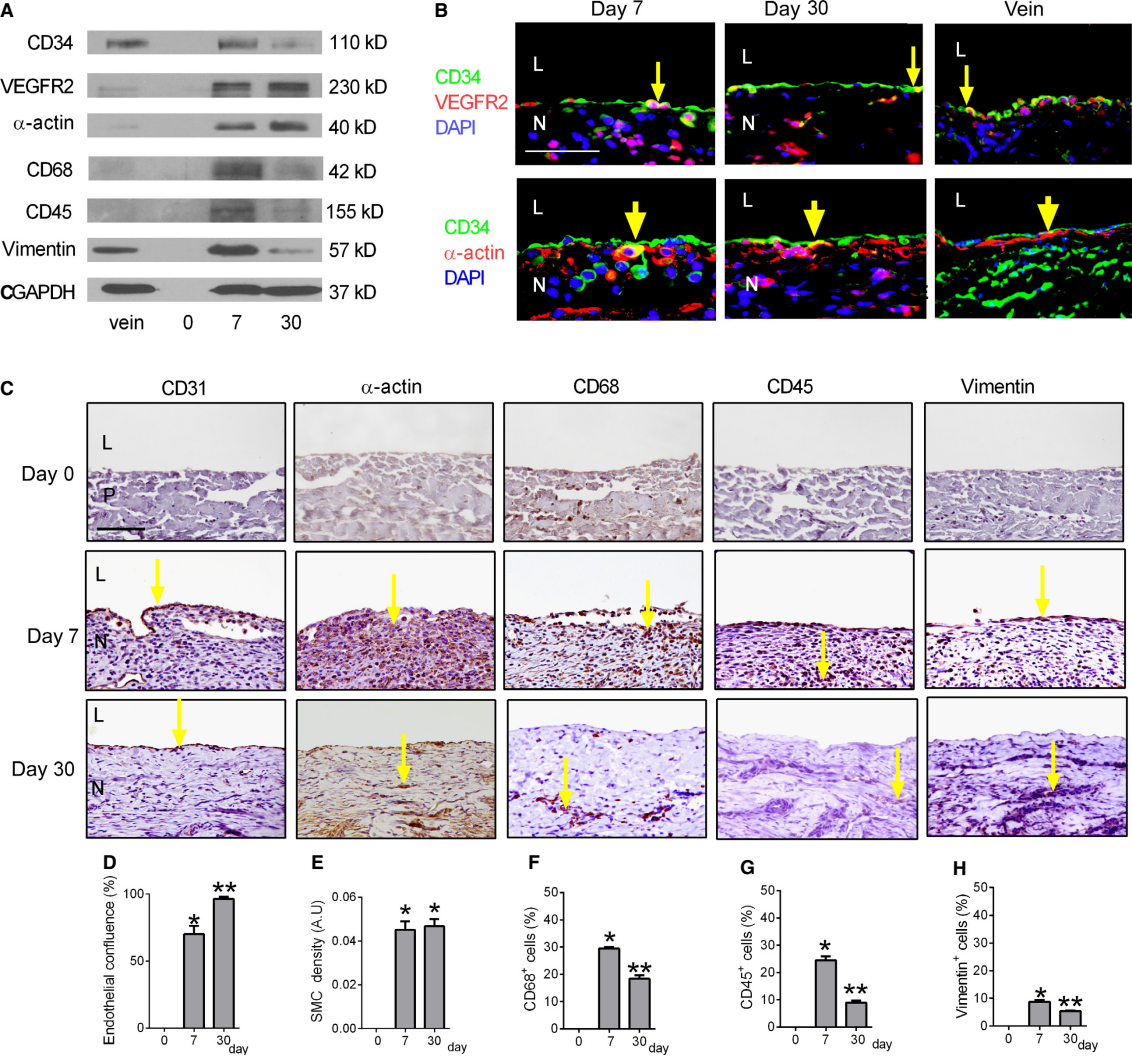
Cell composition of the neointima after patch venoplasty. (A) Representative Western blot showing expression of CD34, VEGFR2, *α*‐actin, CD68, CD45, vimentin and GAPDH in the control IVC (vein), preimplantation patch (day 0), and neointima at day 7 or 30; *n* = 3. (B) Immunofluorescence analysis of the neointima at days 7 or 30 and control IVC (vein). Upper row, merge of CD34 (green), VEGFR2 (red), and DAPI (blue). Lower row, merge of CD34 (green), *α*‐actin (red), and DAPI (blue); scale bar, 50 *μ*m; L, lumen; N, neointima; yellow arrow shows colocalization; *n* = 4. (C) Immunohistochemical analysis of the neointima at day 0 (upper row), day 7 (center row), or day 30 (lower row). Day 0 (upper row) shows control preimplantation patch without neointima. Analysis for: first column, CD31; second column, *α*‐actin; third column, CD68; fourth column, CD45; fifth column, vimentin. P, patch; L, lumen; N, neointima; yellow arrows show the positive cells. Scale bar, 100 *μ*m; *n* = 4. (D) Bar graph showing endothelial confluence; *P* < 0.0001, ANOVA. *P < 0.0001 versus day 0; **P = 0.0048 versus day 7 (post hoc test); *n* = 4. (E) Bar graph showing the mean neointimal smooth muscle cell (SMC) density; *P* < 0.0001, ANOVA. *P < 0.0001 versus day 0 (post hoc test); *n* = 4. (F–H) Bar graphs showing percentage of neointimal CD45, CD68 and vimentin positive cells(mean number of cells counted in 4 high power fields); *P* < 0.0001, ANOVA). *P < 0.0001 versus day 0; **P < 0.01 versus day 7 (post hoc test); *n* = 4.

Since cells form a neointima on the luminal surface of patches placed in the venous circulation, we examined the identity of these cells. Protein from control veins (IVC), the preimplantation patch (day 0), and venous patch neointima at day 7 or day 30 was examined using Western blot; there was expression of CD34, VEGFR2, *α*‐actin, CD68, CD45, and vimentin in the patch neointima after implantation (Fig. 2A). Patch neointimal endothelial cells showed both CD34 and VEGFR2 immunoreactivity at day 7 and day 30, consistent with the presence of endothelial progenitor cells; some cells on the neointimal luminal surface were immunoreactive with both anti‐CD34 and anti‐*α*‐actin antibodies at day 7 and day 30, consistent with smooth muscle progenitor cell identity (Fig. 2B). Control veins without patches showed immunoreactivity with both anti‐CD34 and anti‐VEGFR2 antibodies (Fig. 2B). Cells on the patch neointimal luminal surface stained positively with anti‐CD31 antibody both at day 7 and day 30, consistent with endothelial identity; endothelial confluence, e.g. CD31 positive cells that covered the neointima, was greater at day 30 than day 7 (Fig. 2C and D). Some cells within the neointima stained positively with anti‐*α*‐actin antibody, consistent with smooth muscle cell identity; *α*‐actin‐positive cells generally had a spherical shape at day 7 and a spindle shape at day 30, without any significant difference in neointimal SMC density at days 7 and 30 (Fig. 2C and E). Some of the cells also stained positively with anti‐CD68, anti‐CD45 and vimentin antibodies, consistent with macrophage, leukocyte, and mesenchymal cell identity, respectively; these three types of cells were significantly fewer at day 30 compared to day 7 (Fig. 2C, F, G, H).

**Figure 3:**
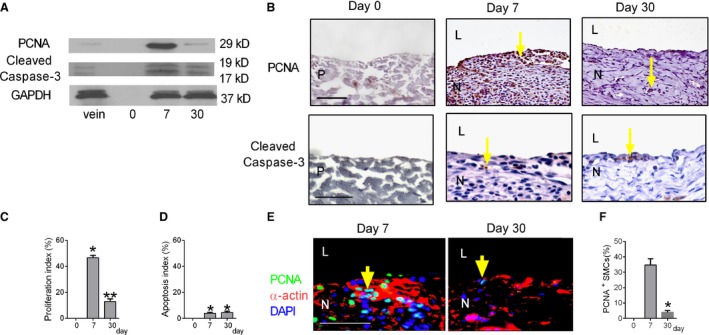
Cell turnover after patch venoplasty. (A) Representative Western blot showing expression of PCNA, cleaved caspase‐3 and GAPDH in the control IVC (vein), preimplantation patch (day 0), and patch explanted at day 7 or day 30; *n* = 3. (B) Immunohistochemical analysis of the neointima at day 0 (left column), day 7 (middle column), or day 30 (right column). Analysis for: upper row, proliferating cell nuclear antigen (PCNA) (scale bar, 100 *μ*m); lower row, cleaved caspase‐3 (scale bar, 50 *μ*m). P, patch; L, lumen; N, neointima. Yellow arrow shows the positive cells. *n* = 4. (C) Bar graph showing neointimal proliferation index (mean number of cells counted in 4 high power fields); *P* < 0.0001, ANOVA. *P < 0.001 versus day 0; **P < 0.0001 versus day 7 (post hoc test); *n* = 4. (D) Bar graph showing neointimal apoptosis index (mean number of cells counted in 4 high power fields); *P* = 0.0061, ANOVA. *P = 0.0158, day 7 versus day 0; *P* = 0.0071, day 30 versus day 0 (post hoc test); *n* = 4. (E) Immunofluorescence analysis of the neointima for PCNA (green), *α*‐actin (red), and DAPI (blue); scale bar, 50 *μ*m. L, lumen; N, neointima. Yellow arrow shows the positive cells; *n* = 4. (F) Bar graph showing neointimal PCNA positive smooth muscle cells (mean number of cells counted in 4 high power fields); *P* = 0.0001, ANOVA. *P = 0.0003, day 30 versus day 7 (post hoc test); *n* = 4.

To determine the turnover of the venous patch cells, we examined proliferation and apoptosis. Western blot showed high levels of PCNA expression in the venous patch neointima at day 7 compared to day 30, and similar expression of cleaved caspase‐3 at these times (Fig. 3A). Since the sensitivities of these antibodies are different, the number of cells that were immunoreactive for proliferation or apoptosis was examined; a similar pattern of expression was seen using immunohistochemistry (Fig. 3B–D), suggesting an overall increased amount of proliferation compared to apoptosis. Since the venous patch neointima contained many SMC, we determined whether some of the proliferating cells were SMC; some neointimal cells were immunoreactive with both anti‐PCNA and anti‐*α*‐actin antibodies, suggesting SMC proliferation in the neointima (Fig. 3E). The percentage of PCNA‐positive smooth muscle cells at day 7 was around 5 times higher compared to that of day 30 (Fig. 3F). With the very low number of cleaved caspase‐3 cells, colocalization with other cell markers was not successful (data not shown).

### Identity of the venous patch neointima

**Figure 4:**
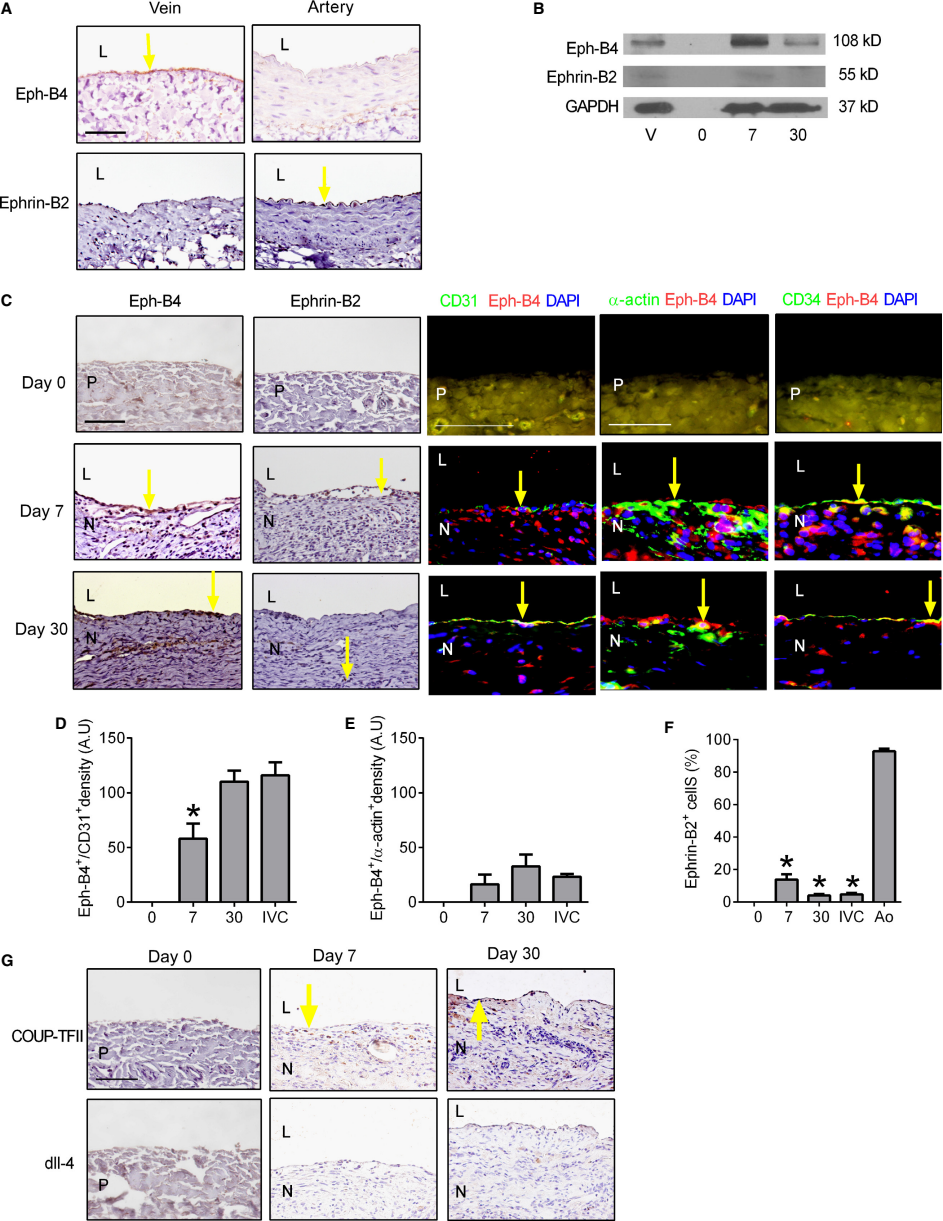
Cell identity after patch venoplasty. (A) Immunohistochemical analysis of rat control IVC (vein) and aorta (artery) for Eph‐B4 (upper row) and Ephrin‐B2 (lower row). Yellow arrow shows Eph‐B4‐positive cells in the vein and Ephrin‐B2‐positive cells in the artery; L, lumen, *n* = 3; scale bar, 100 *μ*m. (B) Representative Western blot showing expression of Eph‐B4, Ephrin‐B2 and GAPDH in the control IVC (vein), preimplantation patch (day 0), and patch explanted at day 7 or day 30; *n* = 3. (C) Immunohistochemistry and immunofluorescence analysis of the neointima at day 0 (upper row), day 7 (middle row), or day 30 (lower row). Day 0 (upper row) shows patch without neointima. Analysis for the following: first column, Eph‐B4 (scale bar, 100 *μ*m); second column, Ephrin‐B2; third column, merge of CD31 (green), Eph‐B4 (red) and DAPI (blue) (scale bar, 100 *μ*m); fourth column, merge of *α*‐actin (green), Eph‐B4 (red) and DAPI (blue) (scale bar, 50 *μ*m); fifth column, merge of CD34 (green), Eph‐B4 (red) and DAPI (blue) (scale bar,50 *μ*m). P, patch; L, lumen; N, neointima. Yellow arrow shows the positive cells. *n* = 3. (D) Bar graph showing density of Eph‐B4 immunofluorescence in neointimal endothelial cells at day 7, day 30 and control IVC;* P* = 0.0002, ANOVA. *P = 0.0316, day 7 versus day 30; *n* = 4. (E) Bar graph showing density of Eph‐B4 immunofluorescence in neointimal smooth muscle cells at day 7, day 30 and control IVC;* P* = 0.0591, ANOVA;* n* = 4. (F) Bar graph showing percentage of Ephrin‐B2‐positive cells in neointima at day 7, day 30, control IVC and control aorta(mean number of cells counted in 4 high power fields); *P* < 0.0001, ANOVA. *P < 0.01; day 7, day 30 and control IVC versus control aorta (post hoc test); *n* = 4. (G) Immunohistochemistry showing the neointima at day 0 (left column), day 7 (second column), or day 30 (third column), stained with COUP‐TFII (upper row) and dll‐4 (lower row), yellow arrow shows the positive cells. *n* = 3.

Since vascular cells infiltrate into the venous patches, we determined whether any of these cells acquire vascular identity. In the embryo, Eph‐B4 is a determinant of venous identity, whereas Ephrin‐B2 is a determinant of artery identity; venous cells express Eph‐B4 both during development as well as in adult veins (Wang et al. 1998). We confirmed that adult rats express Eph‐B4 in the IVC but not in the aorta, and express Ephrin‐B2 in the aorta but not in the IVC (Fig. 4A); accordingly, we hypothesized that the cells that infiltrate into venous patches would acquire venous identity. Western blot showed that control veins express Eph‐B4 and that patches do not express Eph‐B4 at baseline (day 0); however, venous patch neointimal cells expressed Eph‐B4 and low levels of Ephrin‐B2 at days 7 and 30 (Fig. 4B). Immunohistochemistry showed that Eph‐B4 in the venous patch neointima was expressed largely within endothelial cells, similar to the control IVC (Fig. 4C). Immunofluorescence showed CD31 positive cells colocalized with Eph‐B4 both at day 7 and day 30; just below the Eph‐B4 positive endothelial cells, there were some *α*‐actin‐positive cells and CD34 positive cells that also expressed Eph‐B4 both at day 7 and day 30 (Fig. 4C). The Eph‐B4 density that colocalized with endothelial cells increased from day 7 to day 30, with no change in Eph‐B4 density that colocalized with smooth muscle cells (Fig. 4D and E), similar to our previous findings in veins (Muto et al. 2011). Only few neointimal cells expressed Ephrin‐B2 at day 7 or day 30 (Fig. 4C and F). To confirm these data showing acquisition of venous identity, we also assessed COUP‐TFII, another marker of venous identity, which showed immunoreactivity in the venous patch neointima at days 7 and 30; however, dll‐4, another marker of arterial identity, was not reactive (Fig. 4G). These results are consistent with the cells that infiltrate into venous patches acquiring venous identity.

### Early thickening of venous patch neointima compared to arterial patch neointima

**Figure 5:**
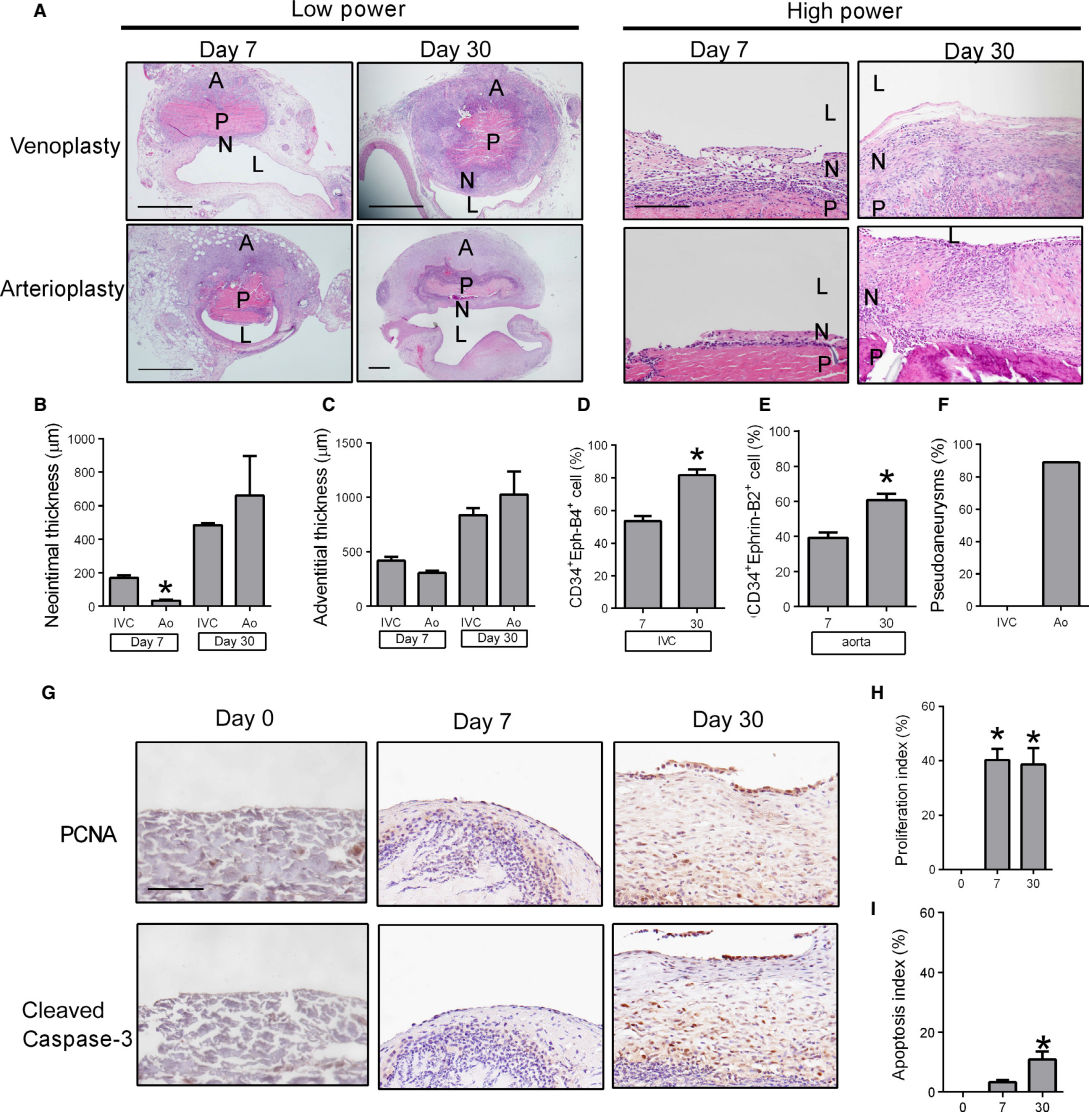
Comparison of patch venoplasty and arterioplasty. (A) Comparison of patches harvested postoperative day 7 or 30 (*n* = 4) after venoplasty or arterioplasty (H&E staining); P, patch; L, IVC or aorta lumen; N, neointima; A, adventitia; lower power scale bar, 1 mm; high power scale bar, 200 *μ*m. (B) Bar graph showing increased neointimal thickness after venoplasty or arterioplasty. *P = 0.0011 versus IVC (*t*‐test); *n* = 4. (C) Bar graph showing increased adventitia thickness after venoplasty or arterioplasty. *n* = 4. (D) Bar graph showing increased neointimal CD34^+^Eph‐B4^+^ cell percentage after venoplasty; *P = 0.0111 (*t*‐test), *n* = 4. (E) Bar graph showing increased neointimal CD34^+^Ephrin‐B2^+^ cell percentage after arterioplasty; *P = 0.0037 (*t*‐test), *n* = 4. (F) Bar graph shows the percentage of pseudoaneurysm formation around the IVC or aorta patches (*n* = 9). (G) Immunohistochemistry showing neointima at day 0 (left column), day 7 (middle column), or day 30 (right column); analysis for: upper row, proliferating cell nuclear antigen (PCNA); lower row, cleaved caspase‐3. P, patch; L, lumen; N, neointima. *n* = 4. Scale bar, 100 *μ*m. (H) Bar graph showing neointimal proliferation index; *P* = 0.0009 (ANOVA). *P = 0.0013 (day 7, post hoc), *P* = 0.0017 (day 30, post hoc); *n* = 4. (I) Bar graph showing neointimal apoptosis index; *P* = 0.0064 (ANOVA). *P = 0.0295 (post hoc); *n* = 4.

Since low flow environments are typically associated with increased neointimal formation compared to higher flow environments (Kohler et al. 1991; Mattsson et al. 1997), we compared the time course of patch neointima in the venous environment compared to similar patches placed in the arterial environment. In the venous environment there was thick neointima formation on the luminal surface of the patch, both at day 7 and day 30; however, in the arterial environment there was a significantly thinner neointima at day 7 with a similar thickness at day 30 compared to the venous environment (Fig. 5A and B). There was no difference in the thickness of the adventitia that formed on the peritoneal surface of the patch in the venous and arterial environments (Fig. 5C). As expected, there were an increased number of CD34^+^Eph‐B4^+^ cells in the neointima from day 7 to day 30 in the venous patches, whereas there were an increased number of CD34^+^Ephrin‐B2^+^ cells in the neointima from day 7 to day 30 in the arterial patches (Fig. 5D and E). There were increased amounts of thrombosis and hemorrhage surrounding the arterial patches at day 30, with increased number of pseudoaneurysms in the arterial patches compared to the venous patches (Fig. 5F). These results are consistent with an earlier thickening of the patches placed in the venous environment compared to those placed in the arterial environment. Interestingly, there was a similar amount of proliferation at day 7 in arterial patches (Fig. 5G–I) compared to venous patches (Fig. 3B–D; *P* = 0.2297, *t*‐test), but arterial patches showed persistent proliferation at day 30 (Fig. 5H; *P* = 0.0160, *t*‐test), consistent with the development of later pseudoaneurysms. There was a similar low amount of apoptosis in the arterial patches compared to venous patches, both at day 7 (*P* = 0.5554, *t*‐test) as well as at day 30 (*P* = 0.0841, *t*‐test).

### Plasticity of the venous patch neointima depends on flow

**Figure 6:**
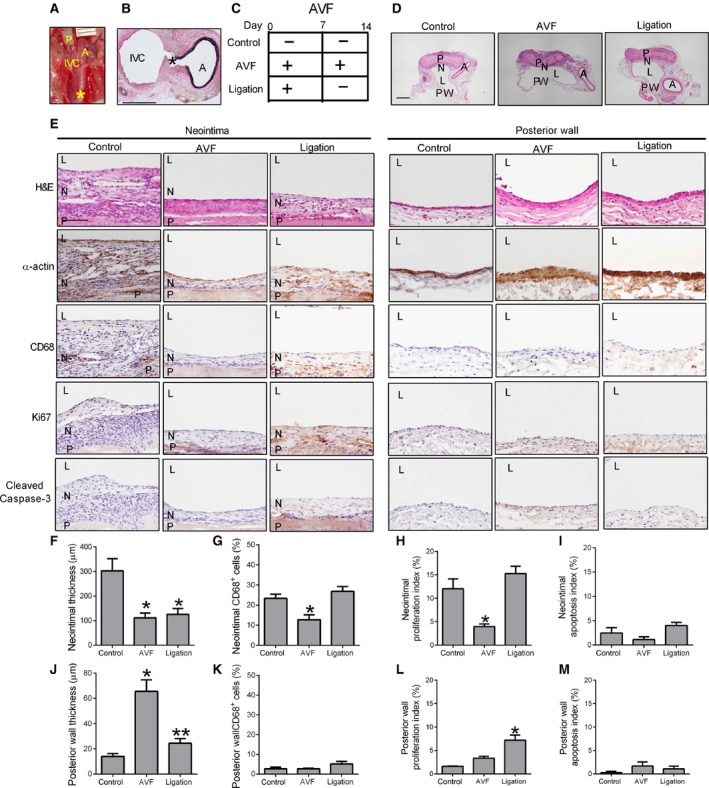
Plasticity of venous neointima thickness with shear stress. (A) Operative photograph showing AVF was created immediately after patch venoplasty. P, patch; A, aorta; IVC, inferior vena cava; *, site of AVF; ruler markings are 1 mm. (B) EVG staining showing the AVF at day 14; A, aorta; IVC, inferior vena cava; *, site of AVF; scale bar, 1 mm. (C) Schematic diagram showing the 3 experimental groups. (D) Representative photomicrographs (low power) showing the different groups of patches harvested at day 14 and stained with H&E; P, patch; N, neointima; L, IVC lumen; PW, posterior wall; scale bar, 1 mm; *n* = 3. (E) Representative photomicrographs (H&E and immunohistochemistry) showing the neointima (left 3 columns) and the posterior vessel wall (right 3 columns), day 14; rows are examined for H&E, *α*‐actin, CD68, Ki67 and cleaved caspase‐3. P, patch; N, neointima; L, IVC lumen; scale bar, 100 *μ*m; *n* = 3. (F) Bar graph showing the mean thickness of the neointima, day 14 (*P* = 0.0246, ANOVA); *P < 0.04 (post hoc test); *n* = 3. (G) Bar graph showing the CD68 positive cell percentage in the neointima, day 14 (cells counted in 4 high power fields), *P* = 0.0116, ANOVA; *P < 0.05 (post hoc test); *n* = 3. (H) Bar graph showing the mean neointimal proliferation index, day 14; *P* = 0.0052, ANOVA; *P < 0.05 (post hoc test); *n* = 3. (I) Bar graph showing the mean neointimal apoptotic index, day 14; *P* = 0.1146, ANOVA;* n* = 3. (J) Bar graph showing the mean thickness of the posterior wall, day 14; *P* = 0.002, ANOVA; *P < 0.01 (post hoc test); *n* = 3. (K) Bar graph showing the mean CD68^+^ cell percentage in the posterior wall, day 14 (cells counted in 4 high power fields), *P* = 0.1568, ANOVA;* n* = 3. (L) Bar graph showing the mean posterior wall proliferation index; *P* = 0.0027, ANOVA; *P < 0.05 (post hoc test); *n* = 3. (M) Bar graph showing the mean posterior wall apoptotic index; *P* = 0.3295, ANOVA;* n* = 3.

Since venous patches develop early neointimal thickening in a low flow venous environment compared to the higher flow arterial environment (Fig. 5), we determined whether venous neointimal thickening is reversible with increased flow. After placing the patch into the venous environment, a distal arteriovenous fistula (AVF) was created via the puncture method as previously described (Fig. 6A) (Yamamoto et al. 2013). AVF remained patent without thrombus formation through day 14 (Fig. 6B and C), resulting in 100% AVF patency and animal survival, with no thrombosis or pseudoaneurysm formation (Fig. 6D). At day 14, venous patches with an AVF show reduced neointimal thickness on the luminal surface of the patch compared to control venous patches without an AVF (Fig. 6C–F); reduced patch neointimal thickness in the presence of the AVF was associated with reduced *α*‐actin and reduced numbers of CD68 cells (Fig. 6E–G). However, venous patches with an AVF showed a thicker posterior vessel wall on the opposite side from the patch, with a similar number of CD68 cells, consistent with the presence of the higher flow in the AVF (Fig. 6D–E and J–K). The thinner venous neointima on the patches with an AVF showed reduced proliferation (day 14) compared to control venous patches without an AVF (Fig. 6E and H); however, proliferation was unchanged in the posterior vessel wall on the opposite side of the patches, with or without an AVF (Fig. 6E and L). Both the neointima on the venous patches and the posterior vessel wall with or without an AVF show similar low rates of apoptosis (Fig. 6E, I, M). These results suggest that venous patch neointimal thickness is reduced in the presence of high flow environments.

To determine if reduced venous neointimal thickening is reversible, the AVF was ligated at day 7 (Fig. 6C). There was no change in neointimal thickness on venous patches that had the AVF ligated compared to patches with continued AVF (Fig. 6D–F); however, there were similar number of CD68 cells in the neointima of patches with the AVF ligated compared to control patches (Fig. 6E and G). Interestingly, the posterior vessel wall opposite to the patch showed less thickness after the AVF was ligated compared to continued presence of the AVF (Fig. 6E and J), but with similarly few number of CD68 cells (Fig. 6K). There was no difference in proliferation in the neointima on venous patches that had the AVF ligated compared to the control patches, but there was an increased proliferation compared to the continued AVF group (Fig. 6E and H). However, the posterior vessel wall on the opposite side of the patches that had the AVF ligated showed a higher proliferation index compared to the control and continued AVF groups (Fig. 6E and L). Both the neointima on venous patches and the posterior vessel wall that had the AVF ligated show a similar low rate of apoptosis compared to the control and continued AVF groups (Fig. 6E, I, M). These results suggest that ligation of the AVF changes cell proliferation, but do not reverse the thinner neointima that forms on venous patches in the presence of the AVF, suggesting that the early venous neointimal formation is established within 1 week.

## Discussion

Using a novel rat patch venoplasty model, we show that pericardial patches implanted into the venous environment show accumulation of cells that are CD34^+^/VEGFR2^+^, e.g. endothelial progenitor cells (Fig. 2), and CD31^+^/Eph‐B4^+^, e.g. venous endothelial cells (Fig. 4), consistent with acquisition of venous identity. We also show that venous patches develop early aggressive neointima hyperplasia compared to the patch arterioplasty model, with no pseudoaneurysm formation (Fig. 5), suggesting that pericardial patches form an environment that promote accumulation of progenitor cells in the absence of arterial pressure, whereas pseudoaneurysm formation after patch angioplasty is an epiphenomenon secondary to arterial environmental factors such as pressure.

The structure of the bovine pericardium patch provides a microenvironment for the attachment, survival, migration, infiltration and/or proliferation of cells, a large advantage over synthetic materials (Chang et al. 2002, 2004). We show that cells accumulated in patches placed into rat veins (Fig. 1), similar to the acumulation of cells in patches placed into rat arteries (Li et al. 2012). It is possible that the ultrastructural features of the collagen in the pericardium forms a physical stimulus to promote the accumulation of cells in the patch, similar to the effects on macrophages (Chen et al. 2010; Bai et al. 2016). It is also possible that the center of the patches are relatively hypoxic, forming another characteristic of the environment that promotes progenitor cell survival (Assi et al. 2016). Regardless of the mechanism, the accumulation of venous progenitor cells in the patch are likely to promote healing and resistance to infection, clinically desireable feastures of pericardial patches (McMillan et al. 2012).

We use the pericardial patch in a novel rat venoplasty model, which has several advantages compared to an arterial model. First, the more aggressive development of neointimal hyperplasia in the venous environment provides a new model of neointima hyperplasia that is easy to perform and analyze, with shorter time to development of stable neointima (Kohler et al. 1991; Shi et al. 1998). Second, the lower pressure of the venous environment does not promote pseudoaneurysm formation (Fig. 5) as commonly seen after rat aortic patch angioplasty (Bai et al. 2016), and human patch angioplasty (Wheeler et al. 2000; Dardik et al. 2001), allowing longer time points for analysis. Third, the patch venoplasty can be combined with other surgical manipulations such as creating a simultaneous AVF to test the effects of increased arterial blood flow on patch neointimal thickness (Fig. 6).

We used an aortocaval AVF to test the effects of arterial blood flow on the thickness of the patch neointima (Fig. 6). The posterior wall of the IVC opposite to the patch showed increased thickness in the presence of the AVF, consistent with adaptive remodeling of veins to the arterial environment (Liang et al. 2013; Yamamoto et al. 2013). However, the neointima on the patch showed decreased thickness in the presence of the AVF, consistent with other models of less neointimal formation in the presence of high flow (Ellenby et al. 1996; Mattsson et al. 1997; Richter et al. 1999; Meyerson et al. 2001; Berceli et al. 2002). Interestingly, ligation of the AVF resulted in reversal of the venous wall adaptive remodeling (Fann et al. 1990), whereas the patch neointimal thickness did not increase (Fig. 6F and J); although patch neointimal thickening may occur at later times, these results suggest that the early aggressive venous neointimal hyperplasia at 1 week (Figs. 1 and 5) is structurally stable. These data also show that venous thickening may be adaptive remodeling or neointimal hyperplasia, and that these processes are distinct from each other.

In summary, pericardial patches placed into the rat IVC show infiltration of cells including macrophages and venous progenitor cells, suggesting that patch venoplasty heals with a venous phenotype and confirming that the patch environment attracts progenitor cell accumulation in the absence of arterial pressure. Venous patches develop neointimal hyperplasia on their luminal surface with more aggressive thickness at 1 week compared to arterial patches, suggesting the utility of this model for studies of neotintimal hyperplasia. Finally, the lack of pseudoaneurysm development in venous patches shows that pseudoaneurysm development in the rat patch angioplasty model is likely to be an epiphenomenon of arterial pressure in this microsurgical model.

## Conflict of Interest

None declared.
